# Human Platelet Lysate-Loaded Poly(ethylene glycol) Hydrogels Induce Stem Cell Chemotaxis *In Vitro*

**DOI:** 10.1021/acs.biomac.1c00573

**Published:** 2021-07-27

**Authors:** Aman S. Chahal, Manuel Gómez-Florit, Rui M. A. Domingues, Manuela E. Gomes, Hanna Tiainen

**Affiliations:** † Department of Biomaterials, Institute of Clinical Dentistry, University of Oslo, Geitmyrsveien 69-71, 0455 Oslo, Norway; ‡ 3B’s Research Group, I3Bs—Research Institute on Biomaterials, Biodegradables and Biomimetics, University of Minho, Headquarters of the European Institute of Excellence on Tissue Engineering and Regenerative Medicine, Avepark—Parque de Ciência e Tecnologia, Zona Industrial da Gandra, Barco, 4805-017 Guimarães, Portugal

## Abstract

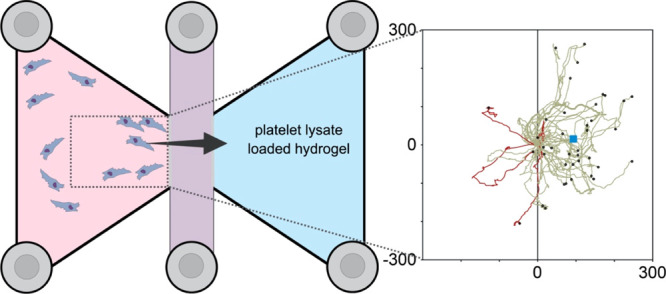

Platelet lysates (PL) contain a selection of proteins and growth factors (GFs) that are known to mediate cell activity. Many of these biomolecules have been identified as chemoattractants with the capacity to induce cell migration. In order to effectively deliver and retain these biomolecules to the site of injury, a scaffold containing PL could be an option. We use poly(ethylene glycol) (PEG) hydrogels consisting of 90 vol % PL to investigate their migratory potential on human mesenchymal stem cells (hMSCs). Cells exposed to these hydrogels were tracked, resulting in cell trajectories and detailed migratory parameters (velocity, Euclidean distance, directness, and forward migration index). Volumetric swelling ratios, hydrogel mechanical properties, and the release kinetics of proteins and GFs from hydrogels were also assessed. Furthermore, hMSC spheroids were encapsulated within the hydrogels to qualitatively assess cell invasion by means of sprouting and disintegration of the spheroid. Cell spheroids encapsulated within the PL-PEG gels exhibited initial outgrowths and eventually colonized the 3D matrix successfully. Results from this study confirmed that hMSCs exhibit directional migration toward the PL-loaded hydrogel with increased velocity and directness, compared to the controls. Overall, the incorporation of PL renders the PEG hydrogel bioactive. This study demonstrates the capacity of PL-loaded hydrogel constructs to attract stem cells for endogenous tissue engineering purposes.

## 1 Introduction

The advancement of regenerative treatments into viable clinical applications is largely limited by the expensive and time-consuming processes of cell isolation and expansion.^1^ Endogenous regenerative therapy is one way of potentially side-stepping this existing hurdle.^2,3^ This involves exploiting the body’s natural ability to recruit stem cells toward the site of injury and directing the cells to repair and restore damaged tissue. In order for this to occur, various growth factors (GFs) are required to stimulate the migration of cells, while supporting their colonization and differentiation at the damage site.^3^ Chemokines that orchestrate the recruitment of stem cells have already been identified and are abundant in blood derivatives such as platelet lysates (PL) and blood serum.^4,5^ Additionally, evidence suggests that a combination of GFs is more effective in recruiting stem cells in comparison to the use of single biomolecules.^6^ While PL has been widely studied to replace fetal bovine serum (FBS) for cell culture,^7,8^ the myriad of GFs within PL has purpose to promote endogenous regeneration as well.^4,9^ Among these GFs, platelet-derived GFs (PDGFs) and vascular endothelial GFs (VEGFs) are important regulators of chemotaxis and angiogenesis during wound healing.^10−12^ Additionally, stromal cell-derived factor-1 (SDF-1α/CXCL12) has widely been recognized as a potent inducer of stem cell migration.^10,13−15^ While the use of platelet-derived products has shown some positive clinical outcomes, the poor characterization, low mechanical competence, and burst release of bioactive molecules from platelet-derived biomaterials have limited their clinical translation.^5,16^

In native tissues, GFs are sequestered, released, activated, and presented by the extracellular matrix, regulating their effects on target cells.^17^ Inspired by this, different biomaterials have been engineered to support these mechanisms, which have shown to drastically potentiate the effects of platelet-derived biomaterials.^5^ Various strategies, including the use of membranes, microspheres, and hydrogels, have been developed for controlled release of these bioactive molecules.^18−20^ Apart from prolonging the release of GFs, such biomaterials can act as a provisional matrix that supports cell colonization and subsequent tissue regeneration.^5,17^ Although the incorporation of PL into tissue-engineered scaffolds is known to enhance cell proliferation, differentiation, and angiogenesis,^21−23^ its capacity to induce the migration of stem cells has not yet been studied extensively, with only limited work highlighting its chemotactic potential.^23,24^

In this study, we explore the concept of a bioactive hydrogel with the capacity to attract stem cells, while remaining mechanically robust to support tissue formation. For this, we produced cell-degradable poly(ethylene glycol) (PEG) hydrogels that comprise 90 vol % PL. As a result, we exploit PEG-based hydrogels to serve as reservoirs of GF-rich human PL while providing a sustained release of proteins. The main aim of this study was to evaluate the effect of proteins and GFs released from the PL-loaded hydrogels on the chemoattraction and migration of human mesenchymal stem cells (hMSCs) *in vitro*. Furthermore, we loaded PEG hydrogels with SDF-1α to benchmark the migratory effects observed in cells exposed to PL-loaded hydrogels against this potent stem cell chemoattractant. This involved a comprehensive assessment of key migration parameters extracted *via* live time-lapse microscopy. In order to study the dynamics of GF and protein release from the loaded hydrogels, BCA analysis along with a multiplex immunoassay was employed. Additionally, due to the abundance of proteins, any interferences imposed by PL on the bulk properties of the hydrogel were assessed *via* rheometry and swelling ratio measurements. Finally, stem cell spheroids were encapsulated within the different hydrogels in order to qualitatively assess whether cells were able to migrate outward and colonize the MMP-cleavable hydrogel matrix in 3D.

## 2 Materials and Methods

### 2.1 Platelet Lysate Isolation and Preparation

Platelet concentrates were obtained under protocols established with Hospital São João (Porto, Portugal). These protocols were approved by the respective hospital ethical committee and according to Portuguese legislation. Any concentrates with platelet counts below 1 million/μL were rejected from the sample pool. The final platelet stock consisted of concentrates obtained from 12 single-donor buffy coats. Platelet concentrates were frozen immediately before or right after expiration, according to protocols established at the hospital. Stocks underwent three rapid freeze–thaw cycles in liquid nitrogen and a 37 °C water bath to lyse the platelets. Finally, aliquots were stored at −80 °C until used. No heparin was used in the platelet lysate stocks. In preparation for experiments, lysed aliquots were thawed and centrifuged at 4000*g* for 5 min and filtered (0.45 μm) to isolate any unwanted debris and clotted fragments. This was performed as previously described.^25,26^

### 2.2 Hydrogel Preparation

Hydrogels were produced from vinylsulfone functionalized 8-arm PEG star macromers (PEG-VS) with a molecular weight of 40 kDa. All polymers were purchased from JenKem Technology USA. Polymers were dissolved either in PL or in serum-free alpha minimum essential media (α-MEM, Sigma-Aldrich, catalog no. 32561). All hydrogels contained 5 wt % polymer. An MMP-cleavable linking peptide (Ac-GCRDVPMSMRGGDRCG-NH_2_) synthesized by Pepmic and dissolved in deionized water (50 mM) was used to end-link the macromers into gels. The same MMP-cleavable linking peptide was used for all hydrogels produced as part of this study. Three sets of gels were generated for this study: PL-loaded (PL-PEG), SDF-1α (SDF1α-PEG)-loaded, and serum-free α-MEM (MEM-PEG) PEG-VS hydrogels. Only MEM-PEG and SDF1α-PEG gels were functionalized with 2.5 mM cRGD [Cyclo(RGD(dF)C), AnaSpec, Fremont, CA]. All SDF1α-PEG gels were loaded with 250 ng/mL SDF-1α. No RGD was added to the PL-PEG gels. PL-PEG gels contained 90 vol % PL solution and 10 vol % of end-linker solution. To produce uniformly shaped gel disks, polymer–end-linker mixtures were pipetted between two glass slides (coated with Sigmacote, Sigma-Aldrich) separated using a 1 mm spacer and allowed to react at 37 °C for 30 min. For migration studies, 65 μL of the gel mixture was pipetted directly into the inlets of the chemotaxis μ-chamber slides (ibidi, Germany). Finally, for cell invasion studies, 30 μL of hydrogels was formed into disks with encapsulated cell spheroids.

### 2.3 BCA Analysis

The Pierce BCA protein assay kit (Thermo Scientific, USA) was used to quantify the total protein released from PL-PEG gel disks as per the protocol provided for microplate assays. Hydrogel disks were placed in 700 μL of PBS, which was collected at 1, 2, 4, 24, and 72 h. The collected samples were frozen at −20 °C until the analysis was performed. To assess the amount of total protein in the PL stock solution, PL was diluted at 1:40 and 1:80 in PBS to achieve a concentration range corresponding to the standards. Standards were prepared as per the protocol provided by the manufacturer, and total protein was measured *via* absorbance at 562 nm. Data are plotted as mean ± standard deviation for independent samples for each time point (*n* = 3).

### 2.4 Release Kinetics of SDF-1α

SDF-1α-loaded gel disks were produced containing 250 ng/mL recombinant human SDF-1α (Shenandoah Biotechnology Inc, USA). Gels were placed in 700 μL of PBS, which was collected at 1, 2, 4, 24, and 72 h. The analysis was conducted immediately after collection. The release of SDF-1α from hydrogels was measured using an ELISA kit (Thermo Fisher Scientific, Catalog #EHCXCL12A) as per the protocol provided by the manufacturer. Measurements were obtained *via* absorbance readings at 450 nm, and values were correlated to the standards.

### 2.5 Swelling Ratio Measurements

The swelling ratio was calculated by measuring the ratio between the swollen (*V*_s_) and unswollen (*V*_r_) hydrogel volumes. A buoyancy kit was attached to an analytical scale to determine volume changes. A total of 30 μL of hydrogels was prepared into disks, as previously described, and the volume was measured immediately after gelation. This was followed by volume measurements by weighing the hydrogels in air and after they had been swollen in serum-free αMEM for 1, 2, 4, 48, and 72 h. Five independent replicates for each gel type were prepared from individual polymer aliquots (*n* = 5). All measurements were prepared at room temperature.

Based on the swelling ratio, the mesh size of an ideal swollen polymer network can be estimated as^27^
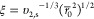
1where υ_2,s_ = 1/*Q*_v_ and  is the mean unperturbed end-to-end distance of the polymer given by
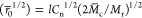
2where *l* is the average bond length (taken as 1.54 Å for vinyl polymers^28^), *C*_n_ is the characteristic ratio (4.0 for PEG^29^), *M̅*_c_ the molecular weight between two cross-links (assumed to be 11,500 g/mol), and *M*_r_ is the molecular weight per repeat unit (44 g/mol). It should be noted that eqs 1 and 2 give a general estimate of the mesh size, assuming an even distribution of cross-linking density, and do not account for network inhomogeneities, such as dangling ends, primary loops, or entrapped entanglements.

### 2.6 Rheology

Large hydrogel discs were prepared and placed on the plate of the rheometer (PP25 on an MCR 301, Anton Paar, Graz, Austria). Oscillatory frequency sweeps were performed at 0.1 and 1% strain, while amplitude sweeps were performed at 1 Hz to confirm that measurements were conducted within the linear viscoelastic regime. Additionally, a normal force of 0.5 N was applied to prevent sample slippage. The presence of cRGD in MEM-PEG and SDF1α-PEG gels was mimicked using *N*-acetylcysteine. All measurements were performed at 37 °C on three independent replicates (*n* = 3). Samples were allowed to swell in serum-free αMEM for two days prior to measurements and were stored in serum-free αMEM during the course of the experiment at room temperature.

### 2.7 Cell Culture

Adipose tissue samples (lipoaspirates) were obtained *via* protocols established with Hospital da Prelada (Porto, Portugal) and approved by the respective hospital ethical committee, according to Portuguese legislation. Tissue samples were processed to isolate adipose-derived hMSCs, according to a previously optimized protocol.^30^ hMSCs used for experimental purposes were cultured (passage ≤ 5) at 37 °C in a 5% CO_2_ incubator using FBS-supplemented α-MEM to maintain cells in an undifferentiated state. Cells used in the experiments were previously characterized using flow cytometry for the expression of mesenchymal stem cell markers, which showed high expression of CD45, CD105, and CD90.^31^ Once cells were 70% confluent, they were trypsinized for seeding into the μ-slide chamber or onto hydrogels. Cells used for experiments were obtained from the same single donor.

### 2.8 Multiplex Immunoassay

Multianalyte profiling using the Luminex 200 system (Luminex, Austin, TX) was conducted to identify and measure the release of GFs from the platelet lysate-loaded hydrogels. As in the BCA analysis, 30 μL of gel discs was placed in 700 μL of PBS, which was collected at 1, 2, 4, 24, and 72 h. The concentration of GFs in each sample was analyzed using the median fluorescent intensity using a 5-parameter logistic line-curve *via* xPONENT 3.1 software. Concentrations of the tumor necrosis factor alpha (TNF-α), C–C motif chemokine ligand 2 (CCL2), C–C motif chemokine ligand 2 (CCL3), epidermal GF (EGF), fibroblast GF 2 (FGF2), interleukin-4 (IL-4), interleukin-8 (IL-8 or CXCL8), VEGF, and platelet lysate GF-AA and -BB (PDGF-AA and PDGF-BB) were measured using a custom analyte human magnetic Luminex assay (R&D Systems, USA). Stock concentrations of each protein or GF were determined by analyzing the contents of the stock PL solution. This is shown in Table S1 in the Supporting Information. The assay was performed as instructed by the manufacturer. Data are plotted as mean ± standard deviation for independent samples for each time point (*n* = 3). To determine the maximum amount of each protein or GF that could be released, PL was diluted in PBS in the absence of the hydrogel (i.e., 100% release).

The hydrodynamic radii of the released proteins were estimated as the Stokes–Einstein radius for globular proteins based on their molecular weights^32^
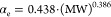
3

### 2.9 Cell Migration Studies and Live Video Microscopy

Cell migration studies were conducted using μ-slides for chemotaxis (ibidi, Germany). Hydrogels were first loaded in the right-most chamber of each three-chambered slide. Gels were allowed to cure for 40 min at room temperature. Cells were then loaded onto to adjacent connecting chamber (1500 cells/mL) in serum-free αMEM. The slide was placed under an inverted microscope with incubation (Axio Observer, Zeiss) equipped with a camera (AxioCam MRm, Zeiss). The camera was programmed to take photos at 10 min intervals for 50 h. Acquired images were exported as a video file and imported into ImageJ for analysis. Videos were first converted to 8-bits to reduce rendering times. A manual tracking plug-in for ImageJ was utilized to generate tracking data from each cell.^33^ For each gel group, 10 cells per chamber were selected at random within a fixed field of view (∼8.3 mm^2^), and cell tracking from four chambers per gel group was used to generate final tracking data (*n* = 40). Tracking data obtained from ImageJ were imported into the Chemotaxis and Migration tool V2.0 provided by ibidi . Cell migration trajectories were generated in relation to the paths adopted by the cells for each group along with shifts in the center of mass. Endpoint parameters were extracted in terms of velocity, directness, Euclidean distance, and the forward migration index (FMI_Δ*x*_). These parameters have previously been defined by Zengel et al. with respect to this particular setup.^34^ Rose plots were generated by transforming the endpoint co-ordinates of each cell trajectory from Cartesian to polar in order to illustrate directionalities adopted by the cells with interior angles set at 22.5° per segment. Cell trajectories are plotted using a coordinate transformation such that all cell paths originated from the same point. Data are plotted as median ± 10th and 90th percentile for graphs, illustrating the migration dynamics.

### 2.10 Cell Invasion Studies

Cell invasion studies were performed by placing a spheroid containing 1 × 10^4^ cells into the gel disk prior to gelation. Spheroids were formed by culturing hMSCs in non-adherent round-bottomed ultra-low cluster well plates (Corning Inc., USA). Once the spheroids had formed in the round-bottomed wells, medium was carefully discarded, and a polymer–end-linker solution was promptly pipetted into the well, such that the spheroid was encapsulated into the solution prior to gelation. This gel mixture containing the spheroid was pipetted between two hydrophobic glass slides to produce a hydrogel disk, as previously described. Hydrogel disks containing the spheroids were placed in 24-well plates containing 700 μL of αMEM supplemented with 10% FBS. Bright field microscopy was used to acquire images of cell spheroids or cells seeded on the surface of hydrogel disks at 1, 3, and 7 d. Representative images from three replicates were acquired for each gel group for 3D invasion studies.

### 2.11 Immunolabeling and Fluorescent Confocal Microscopy

Cells were fixed at day 7 with 4% PFA immediately after culture medium was discarded. Each gel containing cells was submerged in PFA for 30 min. The PFA was discarded, and cells were washed with Dulbecco’s phosphate-buffered saline (DPBS) three times. hMSCs were then permeabilized using 0.1% Triton X-100 for 10 min, followed by a DPBS rinse. Alexa Fluor 568 phalloidin (Thermo Fischer Scientific) solution was prepared using DPBS to obtain working dilutions of 1:400. Cells were incubated for 1 h at room temperature with phalloidin solution for fluorescent labeling of the actin and cytoskeleton. The solution was discarded, and samples were rinsed with DPBS three times. Finally, DAPI was used at 1:1000 working dilution to stain the nuclei. Samples were imaged with a 20×/0.40 HCX PL APO CS objective lens on a Leica SP8 confocal laser scanning microscope using 638 nm excitation for phalloidin and 405 nm for DAPI. Tile scans were performed, and z-stacks were generated where cells in the same field of view did not appear on the same plane due to the multidimensionality of the cell spheroids.

### 2.12 Statistical Analysis

**Figure 1:**
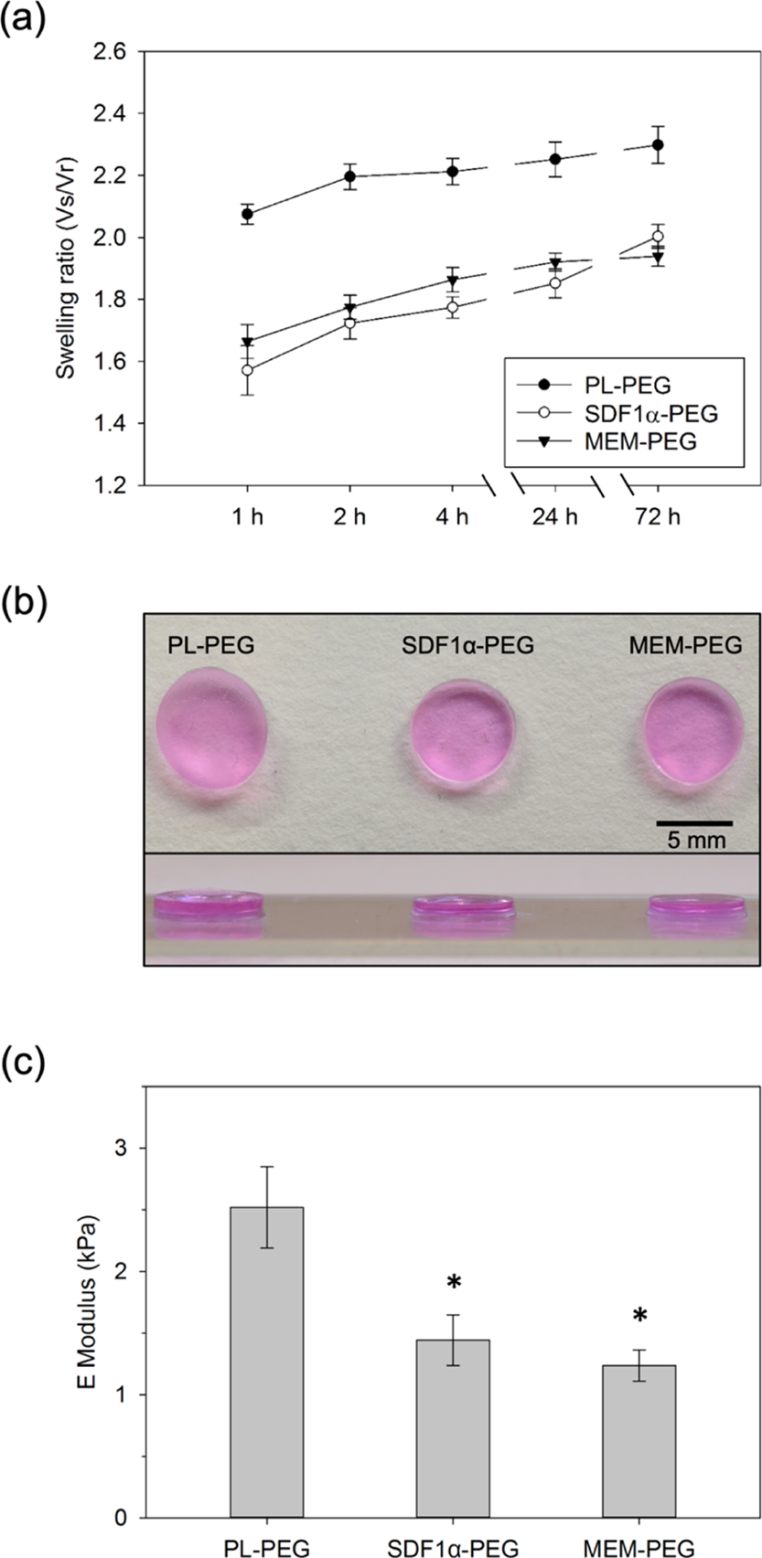
Characterization of hydrogel bulk properties. (a) Swelling ratio measurements of the three different hydrogels groups. Data represent the mean ± SD after swelling in αMEM (*n* = 5). PL-PEG hydrogels exhibited increased swelling at all time points compared to the other two groups (*p* ≤ 0.05). (b) Optical images representing hydrogels swollen in αMEM for 72 h. The average diameters for PL-PEG, SDF1α-PEG, and MEM-PEG gel disks shown in the image are 7.2, 6.7, and 6.6 mm, respectively. The top panel shows a view of the hydrogels from above, while the bottom panel shows a lateral view of the hydrogels. (c) Comparison of storage moduli of all hydrogel groups. (* indicates significance compared to PL-PEG hydrogels where *p* ≤ 0.05).

Statistical analysis was performed using SigmaPlot 13.0 (Systat Software Inc.). Shapiro–Wilk normality tests were conducted. One-way ANOVA tests were conducted using the Tukey test when equal variance tests were a success. However, one-way ANOVA on ranks (Kruskal–Wallis) was implemented when equal variance failed. Statistical significance was considered if *p* ≤ 0.05. Repeated measures of ANOVA with Bonferroni *t*-tests for pairwise comparison were used for analyzing the data presented in Figure 1a. For migration studies, the Rayleigh test was performed on each group to determine the uniformity of the circular distribution (where *p* ≤ 0.05 signifies heterogeneous cell endpoint distributions). All data are plotted as mean ± standard deviation, unless specified otherwise.

## 3 Results

### 3.1 Hydrogel Characterization

Bulk hydrogel properties were assessed *via* swelling ratio measurements and rheometry. The purpose of these tests was to evaluate whether the presence of PL or SDF-1α affected the mechanical properties of the hydrogel or interfered with the gelling capacity of the PEG backbone itself. Swelling ratio measurements were conducted at multiple time points to provide insights into volumetric changes among the hydrogel groups over the course of 72 h (Figure 1a).

PL-PEG gels exhibited a significantly higher volumetric increase compared to the other two hydrogel groups consistently from the initial swelling measurements at 1 h throughout the 72 h period (*p* ≤ 0.05). SDF1α-PEG gels exhibited swelling ratios similar to those seen for MEM-PEG gels. Although SDF1α-PEG gels show higher swelling ratios at the final time point at 72 h in comparison to MEM-PEG gels, no statistically significant difference was found between the two groups at any of these time points. Representative optical images for each group highlight the qualitative increase in gel volume upon swelling at the end of the 72 h period (Figure 1b). PL-PEG hydrogels are noticeably more swollen, both in terms of diameter and thickness compared to the other two hydrogels. However, upon quantification of these parameters, the differences among the gel groups are negligible. Despite the statistically significant difference in the volume swelling ratio measured for the PL-PEG hydrogels compared to that of the SDF-PEG and MEM-PEG hydrogels, the theoretical mesh sizes estimated for the swollen gels were rather similar (PL-PEG: 24.9 nm, SDF-PEG: 23.8 nm, and MEM-PEG: 23.5 nm).

Rheology was conducted to evaluate bulk mechanical changes between the gel groups. Interestingly, the presence of proteins and GFs within the PL-PEG gels increased the bulk stiffness of the hydrogel discs. PL-PEG gels (∼2.5 kPa) exhibited significantly higher E moduli compared to the other two gel groups, while MEM-PEG gels had the lowest stiffness (∼1.2 kPa) of the three gel groups (Figure 1c).

### 3.2 Protein and Growth Factor Release

**Figure 2:**
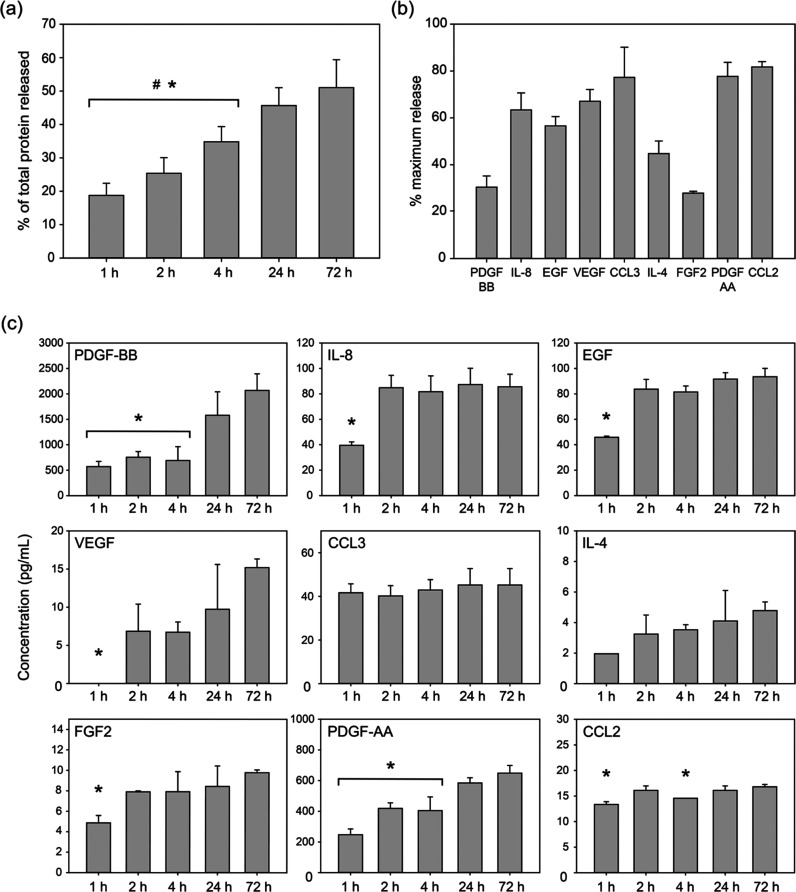
Protein and GF release profiles from PL-loaded PEG hydrogels. All analytes were measured at 1, 2, 4, 24, and 72 h. All data sets are plotted as mean ± SD. (a) Total protein released from PL-loaded PEG hydrogels over the course of 72 h. (*n* = 6). (b) Percentage of GFs released from PL-PEG hydrogels at 72 h (*n* = 3). (c) Concentrations of analytes of interest over the course of 72 h (*n* = 3). # indicates significance compared to samples at 24 h, and * indicates significance compared to samples at 72 h (*p* ≤ 0.05).

Prior to assessing the chemotactic effects of the PL-loaded hydrogels on hMSCs, it was essential to study the protein release kinetic profile from the hydrogels. Hence, BCA analysis was performed to yield the total amount of protein released from the system as a function of time (Figure 2a).

BCA analysis revealed the release dynamics of proteins from the PL-PEG hydrogels over the course of 72 h. One hour after the hydrogels were placed in PBS, nearly 20% of the total protein content had been released. The release kinetics suggested a relatively linear increase up to 72 h, where the total protein content in the surrounding PBS was measured at approximately 50% of the total protein present within the PL-PEG hydrogel (Figure 2a). This suggested a gradual diffusion of proteins from the PL-PEG hydrogels without any external flushing necessary. The release of SDF-1α from the SDF1α-PEG gels was measured using ELISA. Measurements were recorded at the same time points as in the BCA analysis. However, no release was observed at any of the time points (data not shown).

While it is important to confirm that proteins were released from the PL-PEG hydrogels over time, identifying the concentration of each GF within our system is necessary when assessing cellular responses. Hence, a multiplex immunoassay was employed to investigate the contents released from PL-loaded hydrogels. Concentrations of the major components of PL such as PDGF-AA, PDGF-BB, FGF2, EGF, and VEGF were measured. Additionally, other candidate mitogens known to play a role in chemotaxis were also investigated (IL-4, IL-8, CCL2, and CCL3). Transforming GF beta-1 (TGF-β1) was also measured; however, the measurements obtained were below the detection limit of the assay (data not shown).

With more than 80% of the total amount released within 72 h, CCL2 was the GF with the highest release when compared to other GFs measured. However, considerably high amounts of CCL3 and PDGF-AA were also released at 72 h, at 77.7 and 77.2%, respectively (Figure 2b). Interestingly, the alternate isoform of the platelet-derived factor PDGF-BB had significantly lower concentrations compared to PDGF-AA at 72 h. However, FGF2 had the lowest percentage release compared to all the GFs measured. Furthermore, irrespective of the percentage release of each GF, most of the analytes when measured at multiple time points exhibited highest concentrations at 72 h (Figure 2c). Additionally, certain GFs (IL-8, EGF, CCL3, and CCL2) exhibit a high release at 1 or 2 h, followed by a relatively stagnated release. On the other hand, GFs such as PDGF-BB, PDGF-AA, VEGF, and IL-4 were released steadily over time.

### 3.3 Cell Migration

**Figure 3:**
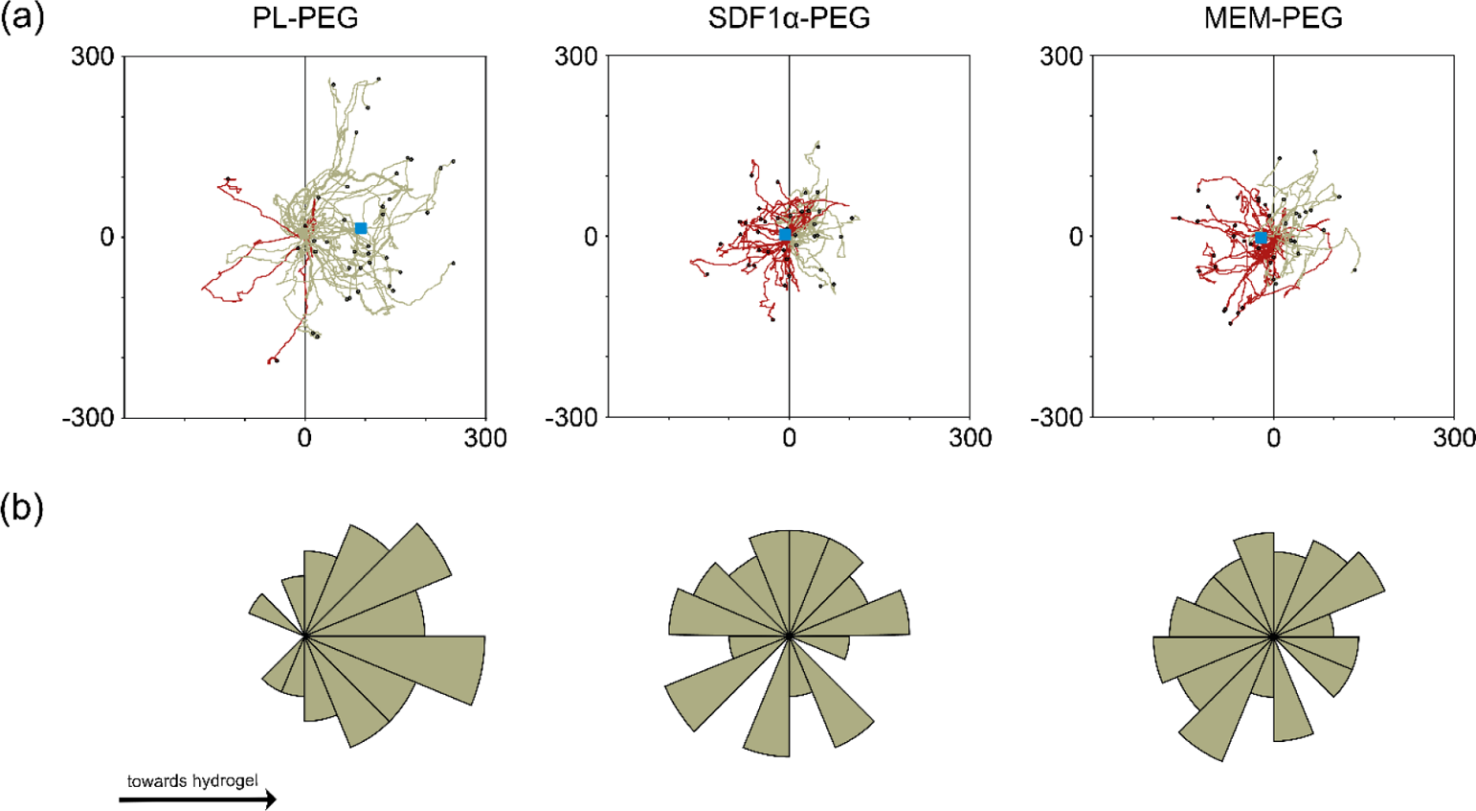
(a) Individual cell trajectories of migrating cells when exposed to gels loaded with PL, SDF-1α, or MEM. The gray line across the *x*-axis (0,*y*) splits the circular positions to the left and right with 180° allocated to each direction. Red lines highlight directions adopted away (left) from the gel, while green lines indicate movements in the direction (right) of the loaded hydrogel. Blue boxes represent the center of mass at the end of the tracking. (b) Rose plots illustrating the distribution of the trajectories at distinct angle intervals. Further extension of segments away from the center represents a larger proportion of cells with endpoints within the defined angle. Additionally, Rayleigh tests were conducted for each hydrogel group to validate the homogeneity of the distribution across the circular plot.

Tracking cells in real time generated trajectories that represented the migratory paths adopted by each cell being monitored. As seen in Figure 3a, 90% of the tracked cells exposed to hydrogels loaded with PL migrated toward the gel, while this was not the case for either SDF-1α (50%)- or MEM (48%)-loaded hydrogels. Instead, cells exposed to these two hydrogels showed no preferred path and migrated to the left and right of their initial point in fairly equal proportion. The shift in center of mass, represented by blue squares in Figure 3a, considers the mean final position of cells at the end of migration. This parameter confirms that most of the cells exposed to PL-loaded hydrogels ended up closer to the loaded hydrogel (right), while cells exposed to the other two gel groups had virtually no shift in their center of mass. The rose plots presented in Figure 3b highlight the distributions of migrating cells at distinct angle intervals. These data complement the observations from the cell trajectory analysis, verifying that hMSCs migrate in the direction of the PL-loaded hydrogels, while this was not observed for the other two gel groups. Rayleigh tests confirmed that cells exposed to SDF-1α-loaded and MEM-loaded hydrogels were distributed homogenously at their migratory endpoints. In comparison, cells in the PL-PEG setup exhibited significantly less-uniform distribution, indicative of preferential directionality (*p* ≤ 0.05).

**Figure 4:**
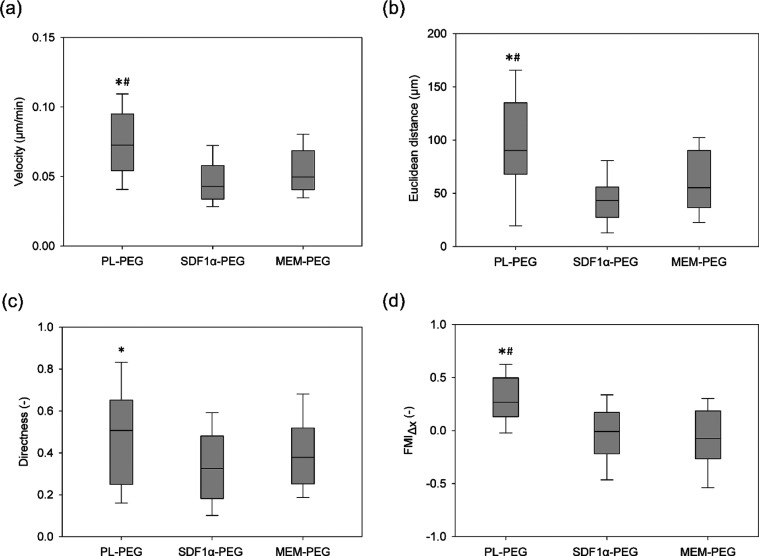
Migration parameters quantified from cell trajectories. Velocity (displacement/time), Euclidean distance, directness (displacement/total path length), and the forward migration index (FMI_Δ*x*_) toward the hydrogel were quantified (*n* = 40). The central line within the box plot represents the median, while the bottom and top whiskers indicate the 10th and 90th percentile, respectively. Cells exposed to PL hydrogels exhibited significantly higher velocities, while covering more distances in the direction of the hydrogel compared to those of SDF-1α (*)- and MEM (#)-loaded hydrogels (*p* ≤ 0.05).

Furthermore, obtained tracking data were analyzed to assess whether the dynamics of cell migration differed among the groups. Figure 4 presents the results for (a) velocity, (b) Euclidean distance, (c) directness, and (d) FMI_Δ*x*_ of the cells tracked from each group (*n* = 40). This data set revealed that cells migrated with significantly higher velocities, covered further distance, and did so exclusively in the direction of the hydrogels loaded with PL when compared to the other two gel groups (*p* ≤ 0.05). However, significant difference in the directness of migration was only observed between cells exposed to PL-loaded gels and SDF-1α-loaded gels.

### 3.4 Cell Invasion

**Figure 5:**
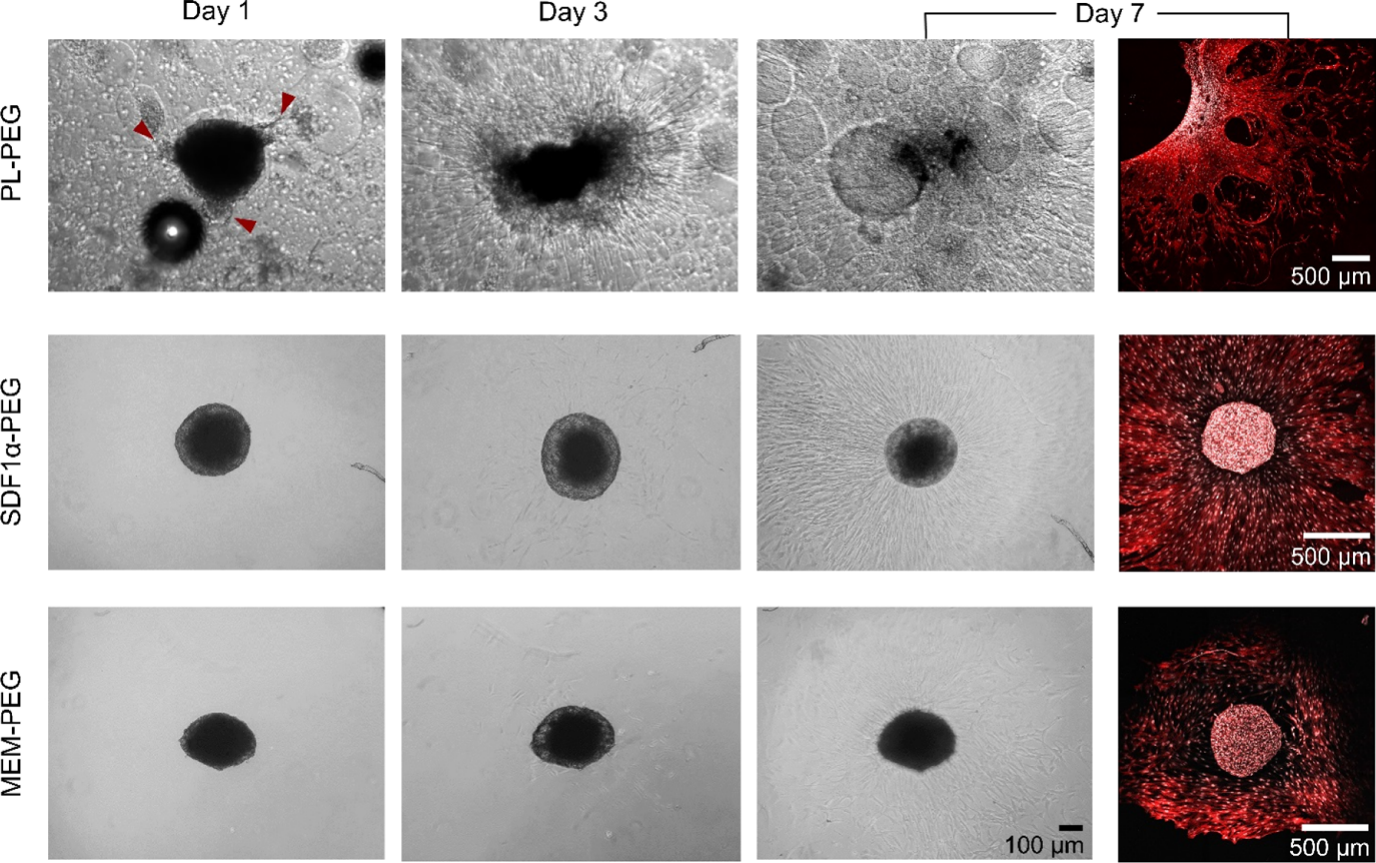
Representative phase contrast and CLSM images of cell spheroids encapsulated within hydrogels for 1, 3, and 7 days. Red arrowheads indicate initial cell invasion at day 1, exclusive to cells encapsulated within PL-PEG hydrogels. Confocal images at day 7 highlight nuclei (white) and actin filaments (red).

In addition to the migration studies, the ability of hMSC spheroids to migrate within the different hydrogels was qualitatively assessed (Figure 5). Cells encapsulated in PL-PEG hydrogels exhibited outgrowths one day after being encapsulated (red arrowheads), whereas this was not observed in either of the two gel groups at this time point. At day 3, images reveal a collapse of the spheroid only in the case of PL-PEG hydrogels. The spheroid continued to disintegrate further at day 7, and cells encapsulated within the PL-PEG hydrogel infiltrated the gel extensively at this point. Only few cells penetrated the hydrogel matrix from spheroids encapsulated within both SDF-1α- and MEM-loaded hydrogels at day 3. Nevertheless, cell colonization increased over time at day 7. However, at these time points, there was no sign of the spheroid collapsing in either of the two hydrogel groups. Confocal laser scanning microscopy (CLSM) images taken at day 7 highlight the extent of infiltration upon labeling of the actin cytoskeleton (red) and the nuclei (white). The extent of invasion for the SDF-1α and MEM hydrogels varied significantly. In some cases, only few cells were found around the spheroid, as shown in the Supporting Information (Figure S1). Additionally, Figure S2 shows that the spheroid and invasive cells were observed on different *z*-planes.

## 4 Discussion

The homing capacity of stem cells is a phenomenon recognized *in vivo* as part of the regenerative and repair processes carried out by these cells.^3^ While stem cells exist in a quiescent state in their niche, GFs are capable of inducing migration in a highly regulated manner.^35^ Endogenous regenerative approaches rely on the prolonged supply of various GFs in order to coax stem cells into a proregenerative state.^3^ PL is not only an attractive natural source of GFs, cytokines and temporary extracellular matrix precursors, and cell-adhesive proteins^5^ but also a clinically viable solution for regenerative medicine, considering its easy production, standardization, and long-term storage.^36^

Endogenous regeneration relies on recruiting cells from adjacent tissues in order to repair damaged tissue.^2,37^ Therefore, a scaffold that supports cell recruitment, colonization, and extracellular matrix remodeling is essential for *de novo* tissue formation. For surrounding cells to migrate toward the scaffold, it is critical that proteins and chemokines are being released in order to direct the migration of the cells. In this regard, we used a protease-sensitive PEG as a backbone, in which PL was incorporated. This system was selected as the hydrogel network to minimize unspecific binding of proteins within PL to the hydrogel backbone,^38^ while allowing cell-mediated hydrogel remodeling. The obtained hydrogels avoided the typical shrinkage of PL gels,^31^ while rendering well-defined, reproducible, and bioactive hydrogels containing 90% protein. In addition, the hydrogel matrix can provide protection against the fast *in situ* proteolytic degradation of PL proteins when delivered to a wound site.

The release of the bioactive molecules from the PL-PEG hydrogels is based on the diffusivity of these proteins through the polymer mesh, making them available to be sensed by surrounding cells. Although approximately, 50% of the total protein was released from the PL-PEG hydrogels over the 72 h period, the results confirm the ability of the PEG backbone to retain and modulate the diffusion of this array of chemokines and GFs. PL-PEG hydrogels also swelled more compared to the other two hydrogels (*p* ≤ 0.05). The higher volumetric swelling of PL-PEG hydrogels suggests that the presence of proteins within PL interferes with the formation of elastically effective chains that cross-link the polymer network. Although this could affect the overall mechanical stability of the hydrogel, the increased swelling capacity facilitates the effective release of biomolecules nonetheless. Concurrently, rheology data shown in Figure 1c suggest that the presence of proteins in PL-PEG hydrogels increased the bulk mechanical stiffness of the hydrogel itself. One explanation for the increased stiffness could be the coagulation of some of the PL components within the cross-linked hydrogel, leading to the formation of an interpenetrating double network. However, this would likely limit the swelling capacity, which was not observed in the case of PL-PEG gels. Whether or not such coagulation occurs, the high concentration of proteins in PL is likely to act as a reinforcing phase within the PEG matrix, increasing the solid content and hindering the movement of PEG chains relative to each other, thus contributing to the increased stiffness of the gel.

Stem cells are known for their ability to secrete and respond to a variety of chemokines and cytokines.^39^ However, the heterogeneity in stem cell populations, both in culture and based on their location in the body, makes it difficult to pinpoint exactly which factors they respond to.^40^ While it is difficult to attribute the directional migration of stem cells observed in this study to a specific set of GFs, the results highlighting the release of a selected panel of GFs may provide some clues (Figure 2a,c). Our subset of analytes was chosen based on their chemotactic role and their presence in PL. Although CCL2 had the highest release (up to 80%) compared to the other GFs, this does not imply that CCL2 solely caused the cells to migrate. In fact, evidence from Ringe et al. suggests that hMSCs do not migrate upon stimulation with CCL2.^41^ The same study, however, identified that CXCL8 (also called as IL-8) has the capacity to stimulate stem cell migration. Additionally, while CCL2 is known to be secreted by stem cells,^42^ its target cell type is often macrophages, and it is therefore also referred to as macrophage inflammatory protein 1α (MIP-1α).^43^ On the other hand, CCL3 has been previously identified as a chemokine capable of attracting bone marrow-derived stem cells.^44^ Given the high amount of release at 72 h in our study, it is possible that CCL3 contributed to the migration of stem cells we observed. However, other vital components of PL might have also contributed to the increased hMSC migration observed in this work.

PDGF, VEGF, EGF, and TGF-β1 are typically among the most abundant GFs of lysed platelets.^4,45^ Our findings of PL-induced hMSC migration are in accordance with those of studies where scaffolds coated with human PL resulted in enhanced migration toward the scaffold.^24^ Additionally, there is growing evidence that PDGF is a more potent chemoattractant than chemokines belonging to the CXC family.^46,47^ In fact, PDGF has previously been incorporated into electrospun scaffolds with the intent to induce MSC migration and has demonstrated greater migration when compared to scaffolds loaded with a cocktail of CXC chemokines.^46^ Furthermore, PDGF is known to be present at concentrations higher than 10-fold in PL when compared to physiological blood serum levels,^5^ making it a likely candidate responsible for the stem cell chemotaxis we observe. While we were able to detect more than 80 pg/mL EGF released from PL-PEG hydrogels, to our surprise, TGF-β1 was below the detection limit of the multiplex assay. Although TGF-β1 may not be a potent inducer of stem cell migration, there is evidence suggesting its central role in mediating migration of hMSCs *via* N-cadherins in a dose-dependent manner.^48^ VEGF is another chemokine detected *via* the multiplex assay. However, previous studies show that while VEGF is potent in attracting endothelial cells and effectively contributes to neovascularization, the migration of hMSCs is unaffected by its presence.^24^ In an attempt to unveil the chemokines that contribute to our observations, it is important to acknowledge that a large proportion of the literature focusses on the migration of bone marrow-derived mesenchymal stem cells. Since we utilized adipose tissue-derived stem cells, caution must be placed when extrapolating previous findings in light of our observations. Nonetheless, the results presented in Figures 3 and 4 highlight the prospects for PL-loaded scaffolds for cell recruitment in endogenous tissue regeneration applications. While evidence exists for the use of platelet derivatives for cell recruitment,^49^ in this study, we provide details pertaining to the migratory characteristics stem cells adopt when exposed to a hydrogel-based PL delivery system *in vitro*.

Cell migration was examined not only in terms of cell mobility toward the hydrogels but also *via* cell invasion through the hydrogel construct in 3D. Hence, cell spheroids were encapsulated within the hydrogels such that the cells were in direct contact with the proteins and GFs. We were interested in whether the presence of PL would have any added benefits on cell invasion and enhance cell colonization in the 3D matrix of PEG-based hydrogels. The encapsulation of hMSC spheroids resulted in a gradual collapse of the spheroids, while cells colonized the PL-PEG hydrogels (Figure 5). Despite their higher swelling, PL-PEG hydrogels were significantly stiffer compared to the other gel groups, suggesting that the cell spheroids did not collapse as a consequence of the hydrogel chains imposing less physical constraint on the spheroid. Another interesting observation seen only in the case of spheroids within the PL-PEG hydrogel was the immediate establishment of attachment onto the 3D matrix (arrowheads in Figure 5). These outgrowths were absent in spheroids encapsulated in the other two hydrogels. Although SDF1α-PEG and MEM-PEG hydrogels were functionalized with RGD, the naturally occurring cell-adhesive proteins such as laminin, fibronectin, and fibrinogen present in PL^50^ made PL-PEG hydrogels far more attractive to cells in terms of attachment and spreading. PL-PEG hydrogels allowed cells to migrate due to the permissive and degradable nature provided by the MMP-cleavable PEG system and the cell-adhesive properties of proteins from PL. This is in agreement with previous studies where incorporating fibrinogen or fibronectin into PEG hydrogels improved the cell-adhesive properties of the hydrogel.^51,52^ While the cell-adhesive proteins within PL support cell colonization, a variety of cytokines and biomolecules released from our PL-PEG hydrogel system possess the capacity to attract stem cells. Together, these properties better recapitulate the function of the native extracellular matrix to promote endogenous regeneration in cell-free tissue engineering strategies.

As for the invasiveness of cells in both the SDF-1α- and MEM-loaded hydrogels, it is important to note that the confocal images presented in Figure 5 represent maximal projections across the *z*-planes. This must be interpreted with caution since the cells observed around the spheroid in Figure 5 exist on different planes as the spheroid itself (Figure S2). The fact that spheroids in these two groups remained intact may suggest that few cells were able to escape onto the tissue culture plastic and proliferate. This was confirmed *via* cross-sectional confocal microscopy images (white arrowhead, Figure S2). While this provides evidence that the hydrogel matrix supports 3D cell culture with the presence of RGD, it is difficult to extrapolate any benefits regarding the presence of SDF-1α.

Although SDF-1α is considered a potent inducer of stem cell migration,^53−56^ we did not observe migration of stem cells toward the SDF-1α-loaded hydrogels. Quantification of SDF-1α release revealed that no release of this molecule was detected from the hydrogels. This likely explains why MSCs were not attracted to the SDF-1α-loaded hydrogel. There are certain physical and chemical means by which our hydrogel system may have retained SDF-1α. The first is that SDF-1α was simply physically entrapped within the hydrogel mesh, preventing it from diffusing out of the system in concentrations that could be detected. Similar overall swelling ratios and calculated mesh sizes for both PL-PEG and SDF-PEG gels point toward the mesh size not being the limiting factor for the release of SDF-1α from the hydrogel matrix, as release of chemokines and GFs of similar or larger molecular weight and hydrodynamic radius was observed for the PL-PEG hydrogels. For example, the calculated Stokes–Einstein radii (α_e_) for proteins released from PL-PEG gels such VEGF and FGF2 are 2.8 and 2.1 nm, respectively. Given that α_e_ for SDF-1α is 1.7 nm, it is unlikely that the hydrogel mesh physically restricts its release. However, this does not account for other physical or chemical interactions between SDF-1α and the functional groups of the polymer itself that could potentially restrict the release of SDF-1α.

Considering that SDF-1α comprises 68 amino acids,^57^ it is possible for these to favorably interact with the vinylsulfone functional group on the PEG backbone. However, in order for this to be the case, SDF-1α is required to have available cysteine residues to chemically bind to the vinylsulfone. While four of the 68 amino acid residues of SDF-1α are cysteine,^58^ these are highly conserved as with other chemokines belonging to the CXC and CC sub-families of chemokines.^59^ Hence, it is unlikely that SDF-1α was chemically bound to the reactive group of the PEG macromer. Swelling ratio results in Figure 1b indicate the same, highlighting that SDF-1α did not affect the formation of elastically effective chains since the volumetric increases were similar to those observed in MEM-PEG hydrogels. While SDF-1α was retained by the hydrogel matrix, alternate loading methods such as bonding strategies *via* ionic complexes and particulate systems^18^ could facilitate its release from the hydrogel.

## 5 Conclusions and Outlook

The present study demonstrated that biomolecules released from the PL-loaded PEG hydrogel scaffolds are capable of inducing directional migration of hMSCs. We provide insights into cell migration parameters and attribute the observed chemoattractive effects to the release of proteins from the PL-loaded hydrogel. While it remains unclear which GFs contributed to the cell migration, PDGF-BB in particular is a likely candidate due to the high amounts released from the hydrogel and its previously known chemotactic capacity. Although SDF-1α was retained within the hydrogels, this does not disregard it as a potent molecule to drive stem cell migration. Instead, optimization is required to release SDF-1α from the PEG-based hydrogel in order to compare its chemotactic effects to those induced by PL.

Upon quantification of various migration parameters, cells exposed to the PL-loaded hydrogels migrated faster, more directly, and covered more distance than cells exposed to the SDF-1α and MEM other two hydrogel groups. Furthermore, the PL-PEG hydrogels presented in this study successfully support cell colonization in 3D without the use of synthetic cell-adhesive peptides such as RGD. Overall, the data presented in this study highlight the use of PL-loaded synthetic hydrogels as an effective approach to attract human adult stem cells from adjacent tissues and allow the cells to colonize the hydrogel scaffold to facilitate endogenous regeneration.

## Supporting Information Available

Additional CLSM images of spheroids encapsulated within hydrogels and concentrations of GFs of interest within PL stocks and controls (PDF)
