# Supplementary material for: Human Platelet Lysate-Loaded Poly(ethylene glycol) Hydrogels Induce Stem Cell Chemotaxis In Vitro
Source: Biomacromolecules. 2021 Jul 27;22(8):3486–96. doi: 10.1021/acs.biomac.1c00573 (PMC8382254; doi:10.1021/acs.biomac.1c00573)
Supplement: Supplementary file 1 — bm1c00573_si_001.pdf [file bm1c00573_si_001.pdf]

Human platelet lysate-loaded PEG hydrogels induce stem cell chemotaxis *in vitro*

Supporting information

Aman S. Chahal †, Manuel Gómez-Florit ‡, Rui M. A. Domingues ‡, Manuela E. Gomes ‡, Hanna Tiainen †

†Department of Biomaterials, Institute of Clinical Dentistry, University of Oslo, Geitmyrsveien 69-71, 0455 Oslo, Norway.

‡3B's Research Group, I3Bs – Research Institute on Biomaterials, Biodegradables and Biomimetics, University of Minho, Headquarters of the European Institute of Excellence on Tissue Engineering and Regenerative Medicine, Avepark – Parque de Ciência e Tecnologia, Zona Industrial da Gandra, 4805-017 Barco – Guimarães, Portugal

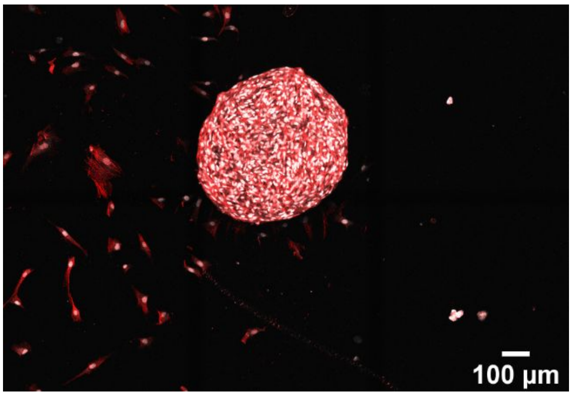

Figure S1: CLSM image of an hMSC spheroid encapsulated within an SDF1α-PEG hydrogel. Actin filaments are shown in red and nuclei in white.

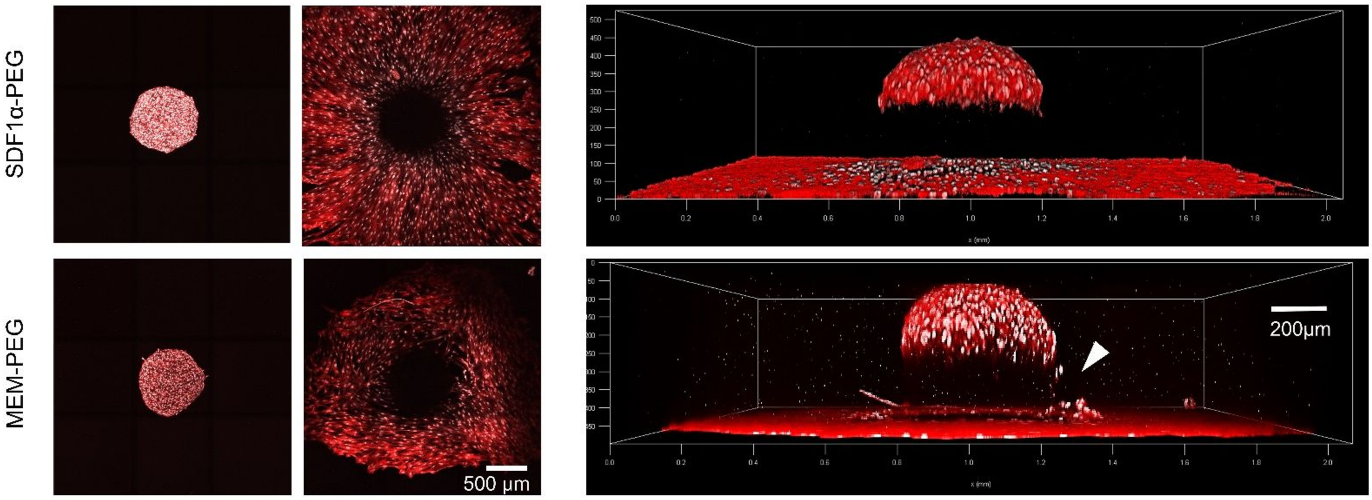

Figure S2: CLSM images of spheroids encapsulated in SDF-1α and MEM loaded hydrogels. Images illustrate that the spheroid and cells around the spheroid exist on different z-planes. Cross-sectional panels on the right show only part of the spheroid, due to focal distance limitations. Cells appear to detach from the spheroid in MEM-PEG hydrogels, indicated by the white arrowhead.

Table S1: Mean concentrations of chemokines in stock platelet lysates. Max release refers to the maximum concentration of each chemokine or growth factor that can be released from each PL-PEG hydrogel. Mean ± SD (n =3). * indicates values that are above the detection limit of the assay.

**Chemokine/Growth Factor**	**Stock Concentration (pg/mL)**	**Max release (100%) (pg/mL)**
PDGF-BB	*	6796.2 ± 2608.4
IL-8	1009.2 ± 384.4	135.0 ± 1.5
EGF	1154.4 ± 646.8	165.1 ± 3.0
VEGF	183.0 ± 34.6	22.6 ± 5.6
CCL3	144.3 ± 6.8	58.5 ± 0.0
IL-4	30.0 ± 3.7	10.6 ± 0.8
FGF2	187.5 ± 19.1	35.1 ± 1.3
PDGF-AA	3078.8 ± 161.5	835.1 ± 92.3
CCL2	161.7 ± 10.6	20.6 ± 0.4
